# Sustainable synthesis of graphene sand composite from waste cooking oil for dye removal

**DOI:** 10.1038/s41598-023-27477-8

**Published:** 2023-02-02

**Authors:** Nor Syazwani Abdullah Sani, Wei Lun Ang, Abdul Wahab Mohammad, Alireza Nouri, Ebrahim Mahmoudi

**Affiliations:** 1 Department of Chemical and Process Engineering, Faculty of Engineering and Built Environment, Universiti Kebangsaan Malaysia, 43600 Bangi, Selangor Malaysia; 2 Centre for Sustainable Process Technology (CESPRO), Faculty of Engineering and Built Environment, Universiti Kebangsaan Malaysia, 43600 Bangi, Selangor Malaysia; 3 Chemical and Water Desalination Engineering Program, College of Engineering, University of Sharjah, 27272 Sharjah, United Arab Emirates

**Keywords:** Environmental sciences, Chemistry

## Abstract

Waste cooking oil (WCO) appears to be a potential carbonaceous source for synthesizing graphene sand composite (GSC) adsorbent in removing pollutants. This study presents a green synthesis method of GSC using WCO as a sustainable carbon source for the synthesis of GSC through the thermal graphitization method. Characterization analysis conducted on GSC_WCO_ verified the successful coating of WCO onto the sand surface and conversion to graphene, which possessed distinct functional groups and features of graphene materials. GSC_WCO_ adsorbent effectiveness in removing Congo Red dye through batch adsorption was studied under the influence of different initial concentrations (20 to 100 mg/L), and the optimum pH (pH 2 to 10), contact time (5 to 240 min), and temperature (25 to 45 °C) were investigated. The GSC_WCO_ showed removal rates of 91.5% achieved at an initial dye concentration of 20 mg L^−1^, 1.0 g of adsorbent dosage, a temperature of 25 °C, and 150 min of contact time. The GSC_WCO_ exhibited a maximum capacity of 5.52 mg g^−1^, was well-fitted to the Freundlich isotherm model with an R^2^ value of 0.989 and had an adsorption mechanism that followed the pseudo-second-order kinetic model. Negative values of enthalpy (ΔH) and Gibbs free energy (ΔG) revealed that CR adsorption onto GSC_WCO_ was a spontaneous and exothermic process. The presence of functional groups on the surface of GSC_WCO_ with such interactions (π–π attractive forces, hydrophobic forces, and hydrogen bonding) was responsible for the anionic dye removal. Regeneration of GSC_WCO_ adsorbent declined after four cycles, possibly due to the chemisorption of dyes with GSC that resulted in inefficient adsorption. Being a waste-to-wealth product, GSC_WCO_ possessed great potential to be used for water treatment and simultaneously benefited the environment through the effort to reduce the excessive discharge of WCO.

## Introduction

Water is one of the most critical components supporting and nourishing the world's ecosystems. The growing urbanization and industrialization activities have significantly affected the ecosystem, releasing pollutants that deteriorate water quality. Hazardous pollutants, such as organic and inorganic materials, heavy metals, dyes, and pharmaceutical residues, are a significant concern to the environment and society due to their toxicity, bioaccumulation, persistence, and non-biodegradable natures^1^. Hence, removing the pollutants and preventing them from being released into the environment have become more appealing and urgent to safeguard the environment. To date, a wide variety of wastewater treatment processes, including adsorption^2^, membrane separation^3^, anodic oxidation^4^, biodegradation^5^, coagulation and flocculation^6^, electrocoagulation^7^, and photocatalysis^8^ have been developed to remove the pollutants following the discharge regulations and to minimize the adverse impact on economic development on the water environment. Adsorption remains one of the preferred processes mentioned above, extensively deployed in the wastewater treatment system. Water utilities' acceptance of the adsorption process could be attributed to its simple, effective, and relatively low-cost operation advantages^9–11^. The success of an adsorption process mainly lies in the adsorbent, which should possess a large surface area or volume, and proper functionalities to capture the pollutants from wastewater^12^.

In recent decades, attention has been shifted to exploring carbon-based nanomaterials as nanosorbents for water purification^13^. One of the fascinating carbon nanomaterials, graphene, has captured the attention of many researchers. Graphene is a monolayer of sp2-hybridized carbon atoms arranged in a 2D honeycomb structure^14^. This material has garnered significant interest owing to its high available surface area, enhanced active sites, and better desorption property^15^. Furthermore, the relatively large and delocalized π-electron system of graphene could strongly influence the binding for target contaminants, making them very attractive and promising as an adsorbent in water treatment. Though graphene displays promising potential as an adsorbent in removing water pollutants, the immediate use of graphene in the actual application is hindered by economic feasibility (large-scale synthesis) and technical difficulties (handling of 2D graphene in the treatment process and separation from treated water for recovery^16,17^. To resolve these obstacles, the facile synthesis of graphene and the immobilization of graphene on proper support for the adsorption process have been explored.

Several efforts have seen the immobilization or coating of graphene on sand (low-cost and readily available material as support) through a facile synthesis approach, where the composites were generally known as graphene sand composite (GSC)^18–21^. These composites were gaining increasing attention as they were easy to synthesize via a simple chemical route, relatively low-cost, and could be produced at a large scale from a diverse carbon source, especially agricultural wastes. Moreover, Dubey et al.^22^ revealed that their GSC could achieve an extremely high adsorption capacity (2859.38 mg/g) for chromium heavy metal pollutants. Such a high removal efficiency was attributed to the presence of charged and electron-donor groups of GSC that reduced the chromium ions and bound them through electrostatic interaction. These results confirmed that GSC effectively adsorbs and removes dissolved pollutants from wastewater. Despite all the advantages, GSC demonstrated some drawbacks in size and particle weight since GSC’s weight is much heavier than the other graphene nanomaterials. Furthermore, the majority of the carbon precursors used for the synthesis of GSC, or similar graphene-supported adsorbent, was sugar, which was coated onto the sand and graphitized to obtain the composite adsorbent. Using sugar as a carbon source is considered non-sustainable and impractical for large-scale synthesis of GSC due to cost factors and risk to food security^23,24^. The exploration of using more environmentally friendly carbonaceous sources for synthesizing GSC has been conducted. Alternative carbon sources such as asphalt^25^, date syrup^26^, palm oil mill effluent^19^, and oil palm frond juice^27^ have been successfully converted to graphene, where the GSC displayed satisfying removal efficiency for a wide range of pollutants.

Given this, waste cooking oil (WCO) appears as a potential low-cost and readily available carbon source for synthesizing GSC. Worldwide, it is estimated that more than 6 million tons of WCO are generated yearly^28^. The improper disposal of WCO is a major environmental pollution problem, and its presence in sewers or drains would lead to blockages and operation issues in wastewater treatment plants^29^. To address these issues, WCO has been utilized as feedstock for various value-added products, including biofuels, plasticizers, binders, surfactants, biomaterials, and different building blocks^30–32^. The valorization of WCO has also been extended to the synthesis of graphene, where the WCO reportedly was successfully converted to graphene via a chemical vapour deposition approach, albeit the information on this topic remains scarce^33,34^. Nonetheless, this suggests that WCO possesses the potential as a carbon source for synthesizing graphene. However, the facile (in-situ) synthesis of GSC from WCO has yet to be investigated. Facile synthesis of GSC from WCO is essential as a complicated synthesis approach will render the economic feasibility of GSC in real applications impractical.

In the presented study, we aimed to develop a novel multifunctional graphene composite from a cheap and locally available waste carbon source, WCO, which acts as a superior adsorbent for removing organic pollutants. WCO will become an excellent alternative to replace other expensive materials owing to its high carbon content and affordable source, which is more favourable for industrial-scale production. In the meantime, further investigation was executed to explore the feasibility of the as-synthesized adsorbent in the adsorptive removal of Congo red dye from an aqueous solution. Morphological and physicochemical analyses were conducted to verify the successful conversion of WCO to graphene. The adsorption kinetic, isotherm and thermodynamics were also determined to provide in-depth insight into the GSC_WCO_ adsorption mechanism. It is believed that the successful anchoring of graphene nanosheets onto superparticles realized via a simple chemical synthesis method can act as an inexpensive material for water pollutant removal. The valorization not only resolves the issues associated with graphene but also helps to minimize environmental pollution from improper disposal of WCO.

## Methodology

### Chemicals and materials

River sand with an average particle size of 0.6–1.2 mm was used to support the immobilization and coating of graphene. The WCO was collected from the café operated on the campus. Sulfuric acid (purity 95–98%) was purchased from R&M Chemicals, whereas Congo red (CR) was obtained from Sigma-Aldrich, Malaysia. All the chemicals used were of analytical grade.

### Synthesis of GSC_WCO_

The river sand was first washed thoroughly with ultra-pure water and dried in an oven. WCO was filtered using filter paper to remove solid objects and precipitate. Next, the WCO was added to sulfuric acid in a ratio of 25:1, continuously stirred, and heated at a temperature range between 120 to 150 °C. The mixture was then mixed with a known amount of sand and stirred for about six hours at a temperature ranging between 80 to 90 °C until the sand was wholly dried and coated with the WCO. Afterwards, the WCO-coated sand was placed in a crucible and heated in a furnace under a nitrogen atmosphere. The furnace was programmed at the temperature as follows:

i. From room temperature to 100 °C in 1 h
ii. From 100 to 200 °C in 1 h
iii. From 200 to 400 °C in 1 h
iv. From 400 to 750 °C in 3 h
v. Held at 750 °C for 2 h (to ensure complete graphitization of WCO).

The black sample produced – GSC_WCO_ was left to cool at room temperature overnight. The GSC was activated by immersing it in concentrated sulfuric acid and kept undisturbed overnight. Lastly, the activated GSC_WCO_ was flushed with ultra-pure water and dried at 100 °C before being used in adsorption testing.

### Adsorption experiment

Batch adsorption experiments were carried out to investigate the effect of various parameters such as initial concentration (20–100 mg/L), adsorbent dosage (0.2–1.2 g), contact time (5–240 min), pH (2–10), temperature (25–45 °C), and ionic strength (0–0.1 M) on the removal of CR. Each experiment was performed by adding 1 g of GSC_WCO_ into 30 ml of synthetic CR solution and agitating in an incubator shaker operating at a constant speed of 150 rpm at 25 °C. The initial pH solution was altered by adding HCl or NaOH (0.1 M). The samples were taken out at a predetermined time, and the absorbance was analyzed with a UV–vis spectrophotometer (DR 3900, Hach, USA) at a wavelength of 496 nm. The residual concentration was calculated from the calibration curve. The removal efficiency (R) and the adsorption uptake at the equilibrium stage (q_e_) were calculated through Eqs. (1) and (2), respectively.

1 $$R\left(\%\right)=\frac{{C}_{o}-{C}_{e}}{{C}_{o}}\times 100\%,$$

2 $${q}_{e}\left(mg/g\right)=\frac{\left({C}_{o}-{C}_{e}\right)V}{M},$$

where ${C}_{o}$ and ${C}_{e}$ are initial and equilibrium concentrations (mg/L) of dye pollutants, respectively, whereas $V$ is the volume of CR solution (L) and $M$ is the mass of GSC_WCO_ (g) used in the study.

Additionally, detailed characterization procedures, including kinetic, isotherm, thermodynamic and regeneration studies, are provided in the Supporting Information (S1–S6).

## Results and discussion

### Characterization of GSC_WCO_

#### Raman analysis

**Figure 1:**
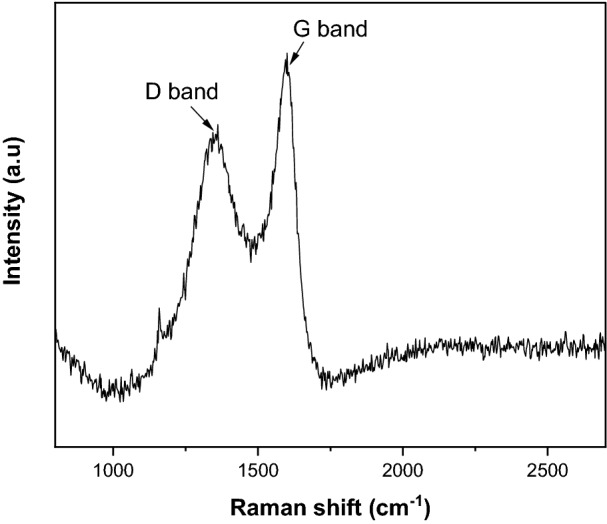
Raman spectra of GSC_WCO_.

Raman spectroscopy was a primary technique used for determining the graphenic forms of carbon. Figure 1 depicts two characteristic peaks of graphene-like structure at 1353 cm^−1^ and 1594 cm^−1^, corresponding to the D and G bands, respectively. The presence of the G band indicates the stretching of C–C bonds between sp^2^-hybridized carbons in graphitic materials, whereas the D band represents defect and disordered graphene structure^35^. The intensity ratio (I_D_/I_G_) of the D and G peaks helps to measure the degree of disorder and crystallite size in the structure of carbon materials. The I_D_/I_G_ ratio of GSC_WCO_ was found to be 0.76, which shows a similar structural disorder as the graphene^36^. On the other hand, no 2D peak was observed between 2500 and 2700 cm^−1^, indicating the formation of amorphous carbon^37^. This postulation follows Gupta et al.^38^ and Ruiz-Hitzky et al.^39^, as it was a standard feature for graphene analogues from a chemically synthesized method. Hence, this result indicated the successful conversion of WCO to graphene composite.

#### X-ray diffraction analysis

**Figure 2:**
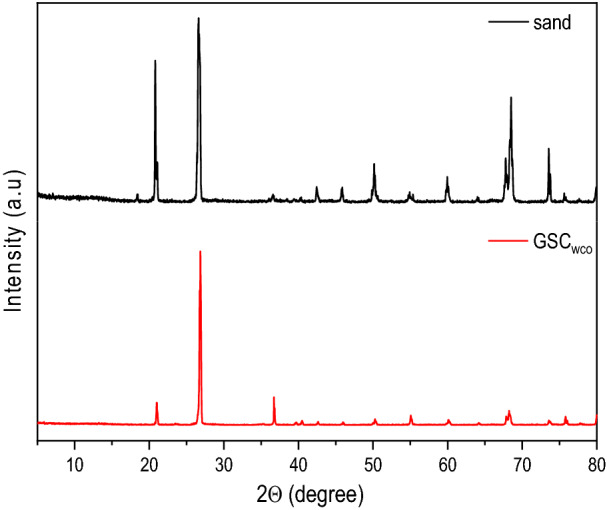
XRD patterns of sand and GSC_WCO_.

XRD pattern of sand and GSC_WCO_ is represented in Fig. 2_._ The sand particles were typically composed of different minerals, such as quartz, kaolinite, calcite, mica, etc. Based on the XRD spectra, the highest peaks of sand can be observed at 26.7° and 20.9° with a crystal plane of (101) and (100), respectively, explicitly referring to the structure of quartz and silica^40^. The XRD spectra of GSC_WCO_ depict a strong peak at 26.8°, which exhibits the characteristic peak of the (002) plane with an interlayer spacing of 3.32 Å. This result reveals the formation of multilayer graphene structures obtained by the successful conversion of WCO on the surface of GSC. The existence of graphene was reflected by the display of strong peaks between the range of 20–27° (2θ), as reported in the literature^16^. In addition, the spectra were seen to have several small and medium diffraction peaks, indicating the presence of minerals from the sand^25^. Similar results were reported by Bajpai et al.^41^, where the trend of diffraction peaks for GSC was found to be lower as compared to sand. This may be attributed to the dispersion of graphene sheets over the sand surfaces that eventually lowered the intensity of the observed peak. The detailed analysis of the (002) plane of GSC_WCO_ is shown in Table 1. The average crystallite size in GSC_WCO_ (34.48 nm) was found to be in the range of crystallinity results obtained from the graphene synthesis using waste cooking palm oil (WCPO) via double thermal chemical vapour deposition (DTCVD) method^42^. This clearly showed that WCO has the potential to be used as a carbonaceous source for the formation of graphene-like carbon materials. The interlayer spacing, crystallized size, and graphene layer were estimated according to the method given in the supplementary information S7.

**Table 1:** XRD analysis detail of carbon peak (002) of GSC_WCO_.

Peak position (2θ, °)	d (Å)	FWHM (β)	Crystallite size (nm)	Graphene layer, n
26.84	3.29	0.23675	34.48	11.48

#### FTIR analysis

**Figure 3:**
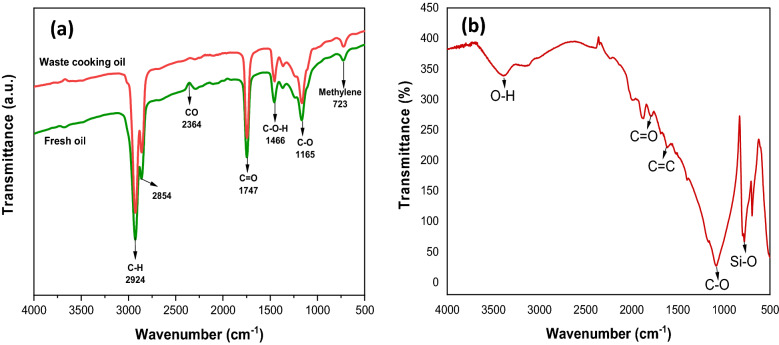
FTIR analysis of (**a**) WCO and fresh oil, (**b**) GSC_WCO_.

The functional groups of WCO and fresh oil were performed using FTIR analysis. Generally, WCO consists of triglycerides, glycerol molecules, a variable quantity of free fatty acids, and several polymerization compounds^43^. As shown in Fig. 3a, both spectra have a similar position and characteristic bands, suggesting the presence of triglycerides component in them^44^. The WCO sample showed an increase in transmittance than fresh oil owing to the existence of FFA, aldehydes, alcohols, and ketones through oxidative and hydrolytic reactions, the consequence of prolonged frying^45^. The absorption bands at 2854 and 2924 cm^−1^ represent the C–H symmetric and asymmetric stretching vibration of aliphatic CH_2_. The peak at 1747 cm^−1^ corresponds to ester C=O stretching of the triglyceride groups. Stretching vibration at 1165 cm^−1^ represents the C–O ester group, while the bands at 1466 and 723 cm^−1^ are associated with the C–H bending vibration of CH_2_ and CH_3_ aliphatic group and overlapping of methylene, respectively^46^. A similar trend was observed in another study by Azam et al.^33^, where an irregular peak was depicted at 2364 cm^−1^ for fresh oil. This indicates the presence of carbon monoxide (CO), which can be considered as impurities that occurs from the exposure of fresh oil to the atmosphere and sunlight.

Figure 3b shows the FTIR spectrum of GSC_WCO_. The band around 3402 cm^−1^ was associated with the O–H stretching of the hydroxyl group. The peak at 1619 cm^−1^ was referred to as aromatic C=C vibrations, which suggests the basic structure of graphene. Meanwhile, the peak at 1794 cm^−1^ confirmed the presence of the carbonyl group in graphene as C=O stretching. It was reported that this observation confirmed the graphitization of WCO on the sand surface^47^. The wide peak at 1080 cm^−1^ was assigned to the stretching mode of the C–O bond^20^. The presence of oxygen functionality in this spectrum was said to help facilitate the adsorption process. The band intensity at 778 cm^−1^ reflects the Si–O stretching mode, indicating the presence of quartz in the sand particles.

#### XPS analysis

**Figure 4:**
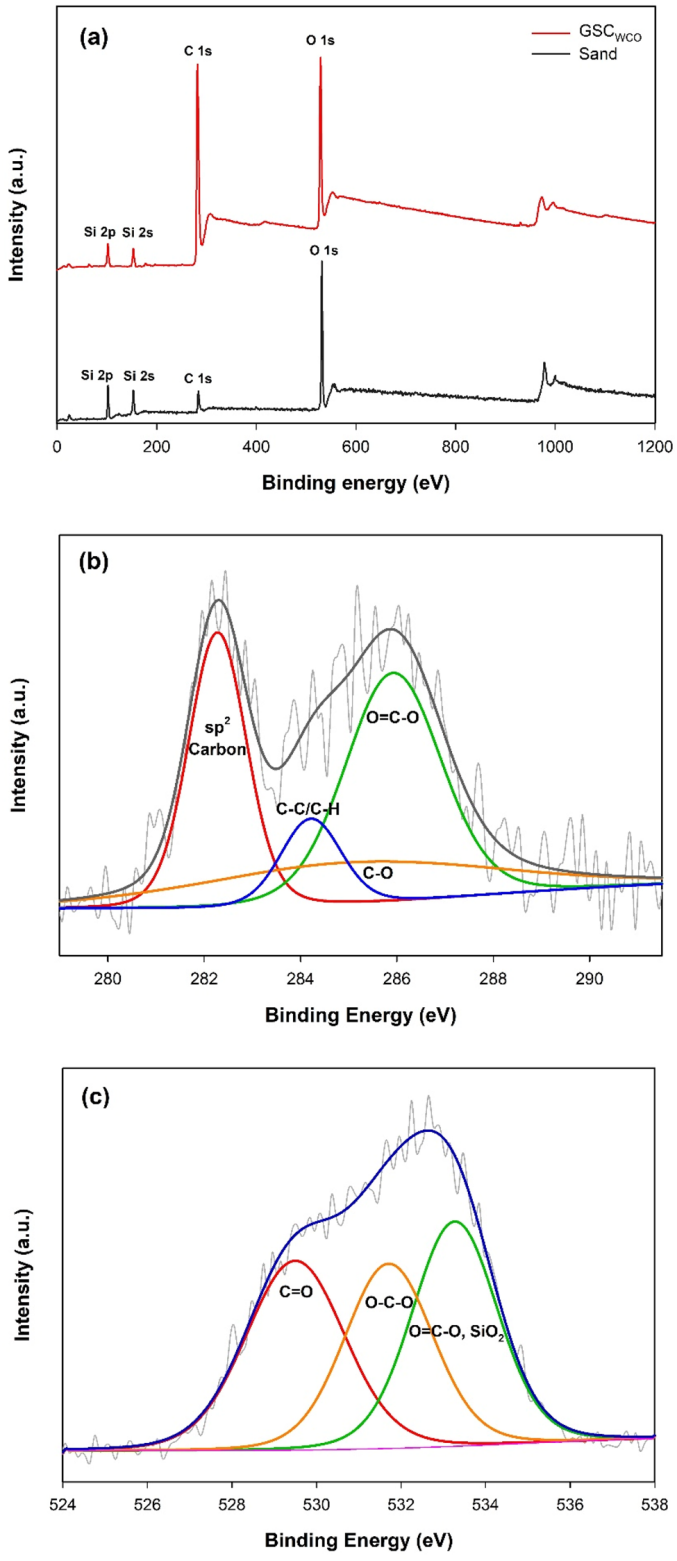
(**a**) XPS survey spectrum of sand and GSC_WCO_, (**b**) high-resolution GSC_WCO_ spectra of C, (**c**) high-resolution GSC_WCO_ spectra of O.

XPS spectrum was used for the elemental analysis of GSC_WCO_. Figure 4a compares the survey pattern of the sand and GSC_WCO_. Four different peaks at 100, 152, 285 and 530 eV related to Si 2p, Si 2s, C 1s and O 1s, respectively, are observed in both patterns. However, after the graphitization of WCO on the sand surface, the silicon content slightly decreased, while there was a significant increase in the carbon content. The illustrated spectra of C 1s in Fig. 4b revealed four prominent carbon peaks of GSC_WCO_. The sp^2^ hybridized carbon has an essential peak at 282.2 eV and other peaks at 284.1, 285.4 and 286.5 attributed to C–C/C–H, C–O and O=C–O, respectively, which verified the successful coating of graphene and oxygen functionalized carbon atoms on the surface of GSC_WCO_. Based on the O 1s spectrum (Fig. 4c), three peaks at 529.1, 531.3, and 533.1 eV exhibited the presence of C=O, O–C–O and O=C–O/SiO_2_, respectively.

#### Scanning electron microscope with electron dispersive X-ray spectroscopy (SEM/EDX)

**Figure 5:**
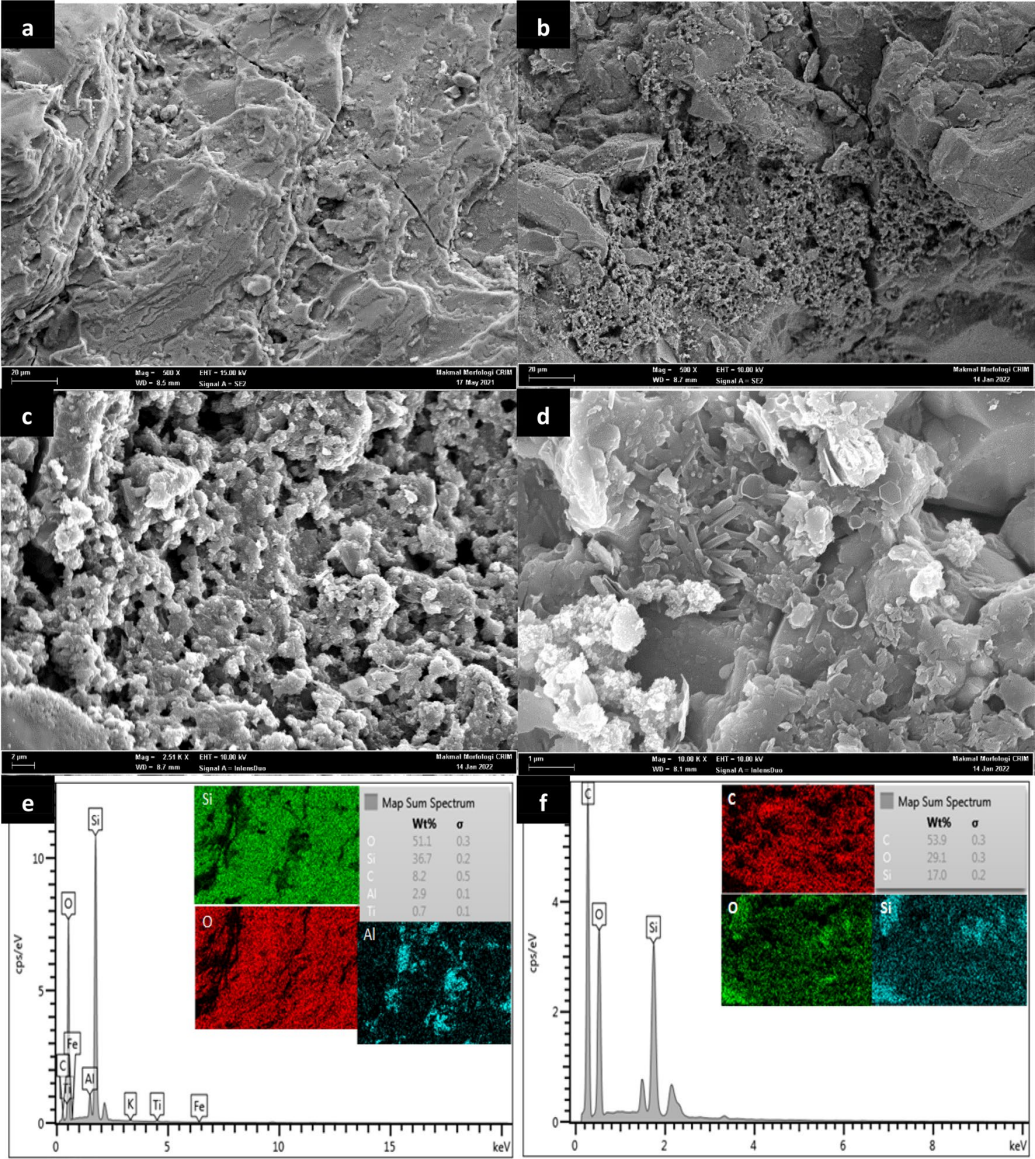
SEM micrograph of (**a**) sand, GSC_WCO_ at (**b**) ×500, (**c**) ×2.5 K, (**d**) CR adsorbed GSC_WCO_ at ×10 K magnification, and EDX/mapping of (**e**) sand, (**f**) GSC_WCO_.

The micrograph of river sand and GSC_WCO_ were investigated by performing SEM analysis. As shown in Fig. 5a, sand had a smoother surface having uniform distribution; however, the structure was slightly rough, indicating the appearance of small fissures on the surfaces^48^. Meanwhile, the surface of GSC_WCO_ (Fig. 5b,c) had quite rough, uneven, and irregular forms with the existence of different pore sizes. Apparently, the development of the GSC_WCO_ porous structure was improved by the activation treatment of the composite with concentrated H_2_SO_4_^18^. The extremely rough and wrinkled sheet-like structure within GSC_WCO_ was said to have more active sites, which can provide stronger adsorption affinity towards pollutant removal. Besides, the WCO coating process was uniformly done as there was the formation of a coating sheets layer covering the sand particles, which was in good agreement with other findings^49^. Similarly, significant changes were observed in the surface of GSC_WCO_ after being adsorbed (Fig. 5d). The pores and external surface of the adsorbent were mostly covered by a thick layer of dye molecules, resulting in a change of its surface morphology (smoother surface with less visible pores). EDX spectrum and the elemental mapping image for the elements that existed on the sand and GSC surface is shown in Fig. 5e,f. The EDX spectra of the as-prepared GSC confirmed the appearance of carbon (C), oxygen (O), and silicon (Si) as major elements. The weight composition of C, O, and Si were found to be 53.9%, 29.1%, and 17%, respectively. As expected, the percentage of carbon content was more dominant in GSC_WCO_ compared to sand and increased remarkably after the coating process, implying the successful coating of WCO and conversion to graphene on the sand surface.

#### Transmission electron microscopy (TEM)

**Figure 6:**
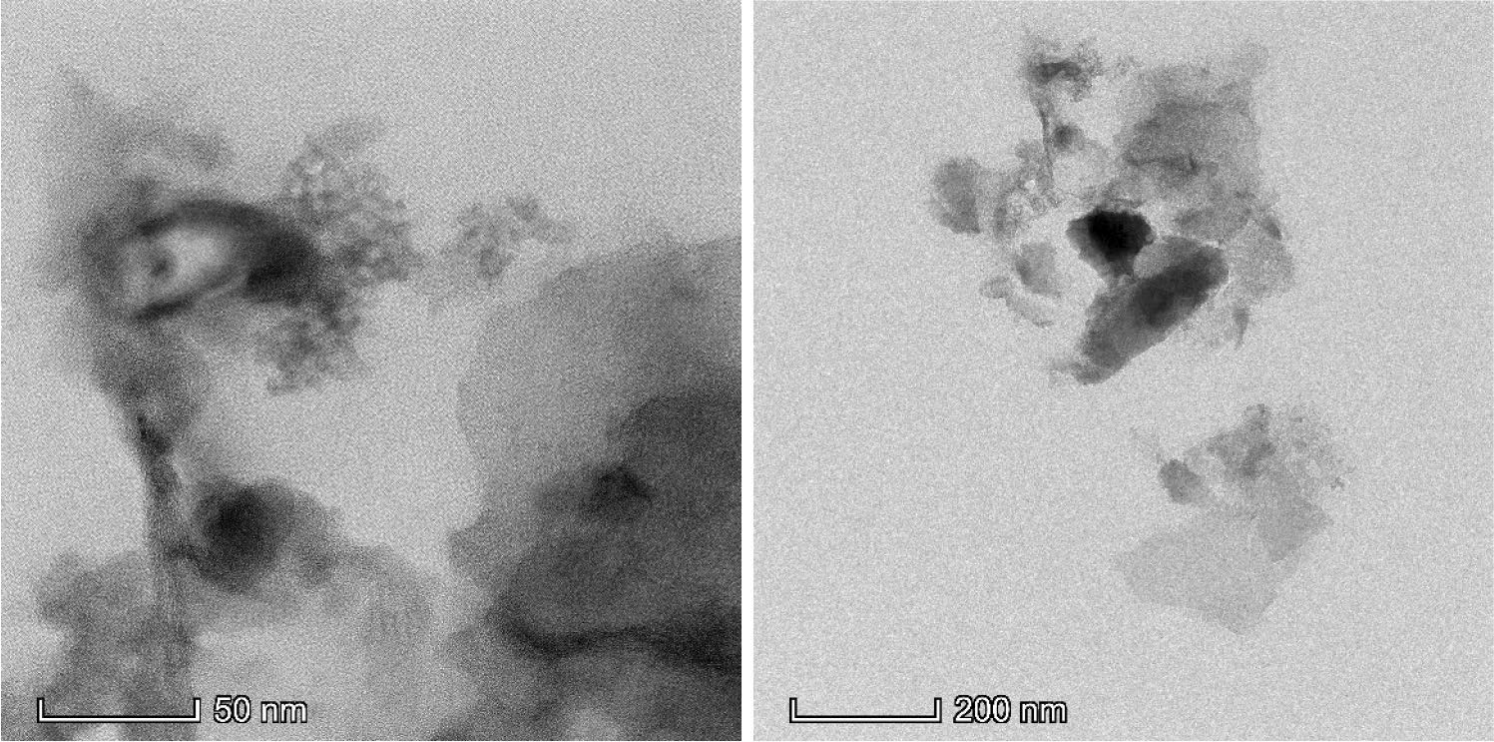
TEM micrographs of GSC_WCO_.

Further confirmation of GSC_WCO_ features and distribution of graphenic material over the sand particles were made through observation with TEM. Figure 6 presents the low-resolution TEM images of GSC_WCO_ features at different magnifications. The image demonstrates a transparent and thin layer sheet with a typical wrinkled structure and corrugation behaviour on the surface of GSC_WCO_, illustrating the multilayer graphene structure. This morphology shows the extent of graphene sheet formation covering the sand surface, which coincides with the SEM results.

#### BET surface area

The BET surface area is one factor influencing the adsorption performance. GSC_WCO_ surface area was relatively small (~ 2 m^2^/g) compared to other reported BET surface areas of nano-sized adsorbents. This lower value indicates that the surface area of GSC_WCO_ was affected mainly by the sand particles, which was in good agreement with the findings of Yang et al.^50^. The graphene sheets on the surface of sand tend to aggregate and re-stacking because of strong π–π bonds and Van der Waals interactions, resulting in a surface area lower than the theoretical value^51^. In addition, since the particle size and weight of sand were much more significant compared to nanomaterials, this could significantly reduce the BET surface area of GSC_WCO_ as the enormous weight of sand led to a smaller surface area. Nevertheless, this finding suggested that the presence of graphene nanosheets having several active sites covering the sand surface has dominated the adsorption capabilities of GSC_WCO_ and was sufficient to remove dye pollutants.

### Adsorption performance

#### Effect of initial concentration

**Figure 7:**
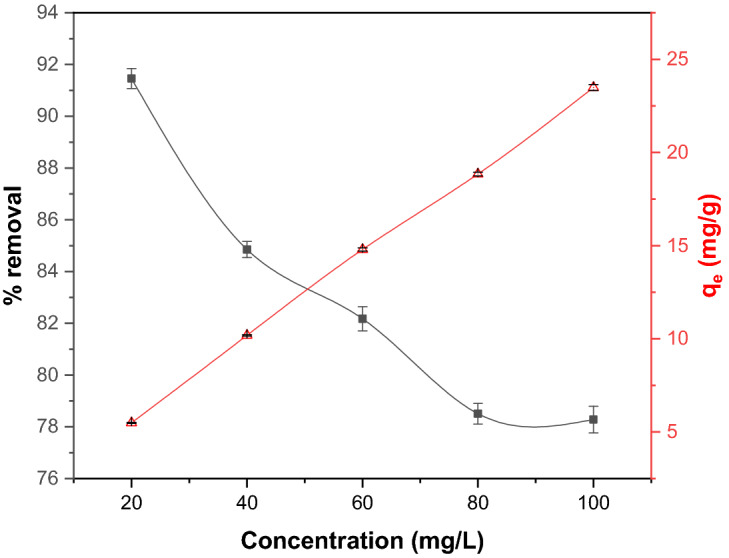
Effect of initial concentration on adsorptive CR removal using GSC_WCO_.

The effect of initial concentration on CR adsorption was studied in the range of 20 to 100 mg/L with constant adsorbent dosage (1 g) at 25 °C. As indicated in Fig. 7, the uptake percentage declined by increasing the pollutant concentration from 91.5 to 78.3%. The highest removal efficiency could be seen at a concentration of 20 mg/L, indicating large amounts of vacant active sites available for CR dye adsorption. However, the percentage removal declines as the CR concentration increases due to the limited adsorption active sites available on the surface of the adsorbent. As demonstrated in Fig. 7, the adsorption capacity increased rapidly at the initial stage with the rise of initial concentration. This behaviour can be explained by the high driving force of mass transfer resistance between the aqueous and solid phases^52,53^. Then, it slowly reached the plateau as the adsorption sites were fully occupied when the concentration of CR was increased. On the other hand, the greater collision between CR molecules and the adsorbent further enhances the adsorption process. This observation is consistent with previously reported findings^54^.

#### Effect of adsorbent dosage

**Figure 8:**
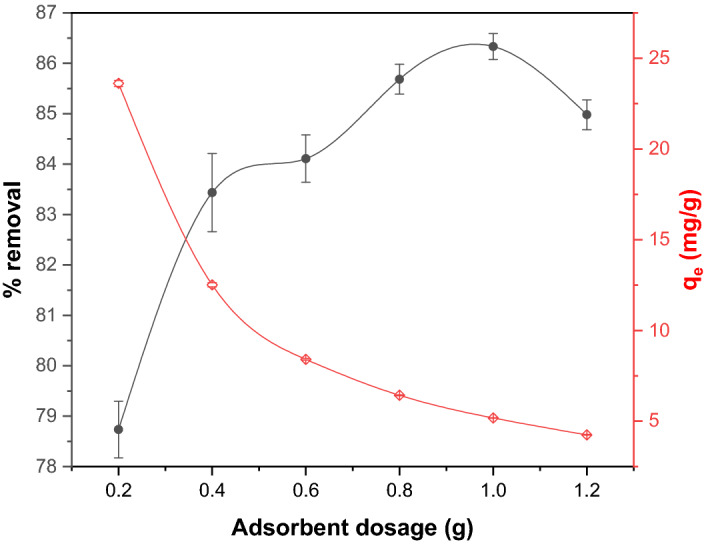
Effect of adsorbent dosage on adsorptive CR removal using GSC_WCO_.

To find the optimized dosage, the experiments were carried out using 0.2 to 1.2 g of adsorbent dosage at a fixed CR concentration of 20 mg/L at 25 °C. The results of adsorbent dosage on adsorption capacity and removal efficiency have been plotted in Fig. 8. The removal efficiency was observed to increase from 78.7 to 86.3% when the amount of GSC_WCO_ dosage increased from 0.2 to 1 g/ 30 ml. This indicates the larger amounts of vacant adsorption sites available for binding and greater surface area for adsoption^55^. The adsorption capacity would reduce its removal ability by increasing the adsorbent dosage beyond the optimum dosage values. The decrease in the adsorption capacity with increased adsorbent dosage was primarily attributed to the overlapping or occurrence of particle aggregation, which could lead to a decrease in surface area and adsorption sites^56,57^. A similar trend was reported by Hou et al.^58^ on CR removal from aqueous solution using chitin suspension. As a result, the optimum dosage of 1 g was chosen due to its high removal efficiency and fixed for the following experiments.

#### Effect of pH

**Figure 9:**
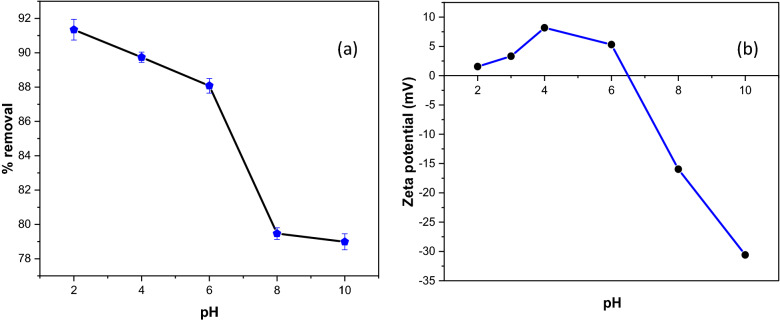
(**a**) Influence of pH on adsorptive CR removal using GSC_WCO_, (**b**) zeta potential of GSC_WCO_ at different pH values.

pH solution is one of the crucial parameters influencing the interaction between the adsorbed molecules and adsorbent particles by affecting the charged nature of functional groups on the adsorbent and the ionization degree of dye pollutants^59^. Figure 9a shows the uptake removal of CR at different pH solutions (pH 2 to pH 10). The dye total uptakes of CR were typically decreased from 91.5 to 79.0% with increasing pH solution. The results imply that CR adsorption on GSC_WCO_ was more favourable in an acidic medium (pH 2). The zeta potential measurements of the GSC_WCO_ composite as a function of pH are displayed in Fig. 9b. From the graph analysis, the GSC_WCO_ attained an isoelectric point at pH 6.5. The positive zeta potential in acidic pH was mainly due to the protonation of amine groups on GSC_WCO_. The predominant charge at the GSC_WCO_ surface was positive at pH < pH_ZPC_ and negative at pH > pH_ZPC_. In an acidic medium, a high amount of hydrogen ion concentration (H^+^) present in the solution protonates the surface of GSC_WCO_. Hence, a significantly strong electrostatic force of attraction occurred between the negatively charged CR molecules (–SO_3_−) and the positively charged adsorbent surface, resulting in a high percentage of dye removal^60,61^. Also, other interactions (π–π bonding) may be one of those factors contributing to the greater removal efficiency. In contrast, anionic CR dye adsorption was less efficient at elevated pH, as there was a competition between excess hydroxyl ion (OH^−^) and CR anions for adsorption sites and the repulsive forces exist between the negatively charged adsorbent, and anionic ions of CR could lead to a decrease in CR removal efficiency. Notably, the amount of CR could still be adsorbed at higher pH owing to the hydrophobic interaction mechanism in the adsorption process. A similar observation was reported for the sorption of CR on mesoporous-activated carbon^62^ and activated carbon-coffee waste^63^.

#### Effect of contact time

**Figure 10:**
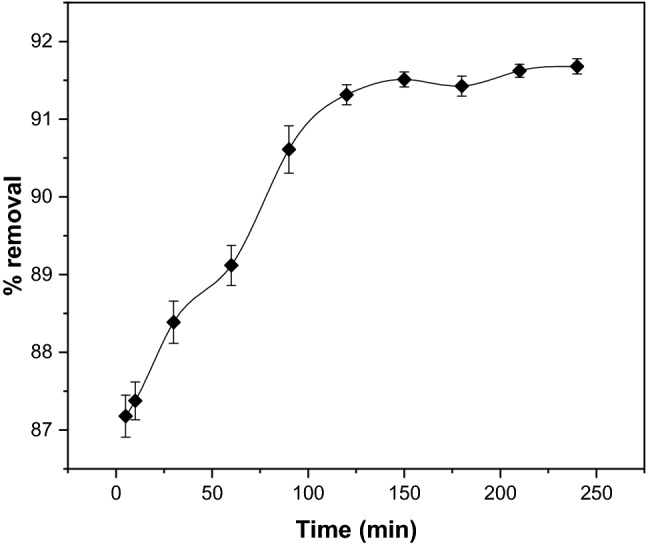
Effect of contact time on adsorptive CR removal using GSC_WCO_.

Contact time is essential as it significantly influences the favourability of the adsorption process. The effect of contact time on the adsorption of CR was carried out in the range of 5 to 240 min under optimized initial concentration (20 mg/L), dosage (1 g), and pH 2. As seen in Fig. 10, the removal trend increased as the contact time increased from 5 to 240 min until equilibrium was reached. It was noticeable that a rapid sorption rate occurred in the early contact period, where nearly 80 to 90% of dye uptakes were adsorbed onto the surface of GSC_WCO_. This was mainly due to the greater number of available vacant sites and the high driving force that eventually helped to speed up the removal rate of CR^64^. The adsorption slightly slows down in the later stages since the exterior surface of the adsorbent has been occupied and become saturated by the molecules of dye. Upon attaining the equilibrium condition, the removal rate could no longer increase as the remaining vacant sites had difficulty occupying the position. This may be ascribed to the repulsive forces between the molecules adsorbed on the surface and the bulk phase^65^. Conclusively, the equilibrium time for CR adsorption was attained within 150 min, where about 91.5% of CR had been removed.

#### Effect of temperature

**Figure 11:**
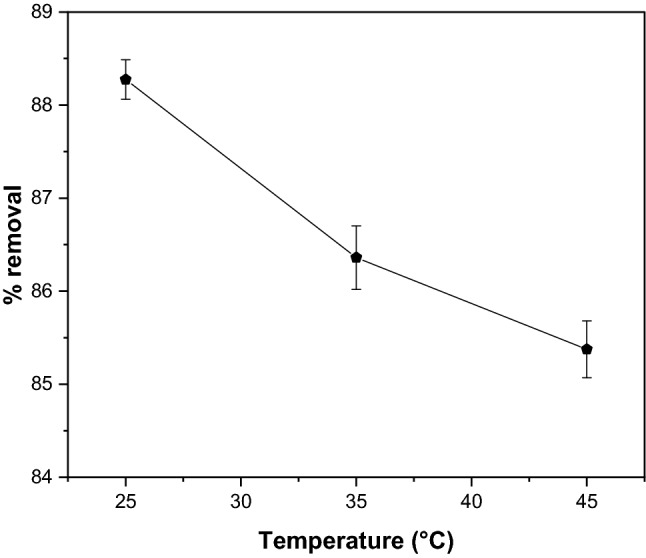
Effect of temperature on adsorptive CR removal using GSC_WCO_.

Temperature is another critical factor influencing the mobility of pollutants and adsorbent properties^66^. In this study, the influence of the temperature of GSC_WCO_ on the elimination of CR was investigated at various temperatures, including 25, 35, and 45 °C with an optimum dosage of 1 g, pH 2, and an initial concentration of 20 mg/L. As shown in Fig. 11, the maximum dye removal was achieved at 25 °C. It was seen that the removal efficiency slightly drops from 88.3 to 85.3% in contrast with the rise of temperature. Our observation aligns well with previously reported work, considering that the decrease in adsorption rate and capacity was inferred as an exothermic process in nature^67^. Two plausible factors contribute to this phenomenon; the first indicates that the physical bonding between the dye molecules and the GSC_WCO_ has weakened, resulting in reduced adsorption as the temperature rises. Another reason may be the increase in dye solubility, which creates stronger solute interaction with solvent^68^.

#### Effect of ionic strength

**Figure 12:**
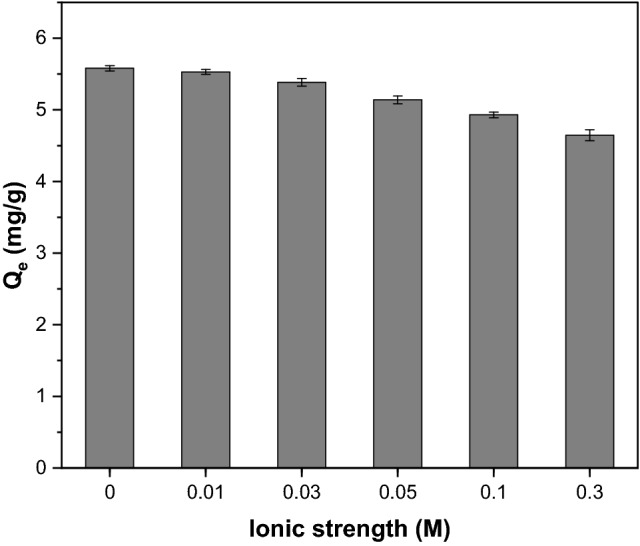
Effect of ionic strength on uptake capacity of CR by using GSC_WCO_.

Figure 12 illustrates the effect of ionic strength on CR uptake capacity onto GSC_WCO_. Ionic strength was adjusted by adding different concentrations of NaCl solution, ranging from 0 to 0.3 mol/L. It was noticeable that there was a slight reduction in the CR uptake capacity from 5.58 to 4.64 mg/g as the NaCl concentration increased. This behaviour was mainly ascribed to the competition between CR and chloride anions (from NaCl) for the available active sites on the GSC_WCO_ surface. The influence of ionic strength was weak, thus, suggesting that the electrostatic interaction was not the dominant mechanism for removing CR on GSC_WCO_. The such postulation was supported by the findings of Liu et al.^69^.

### Isotherm studies

**Figure 13:**
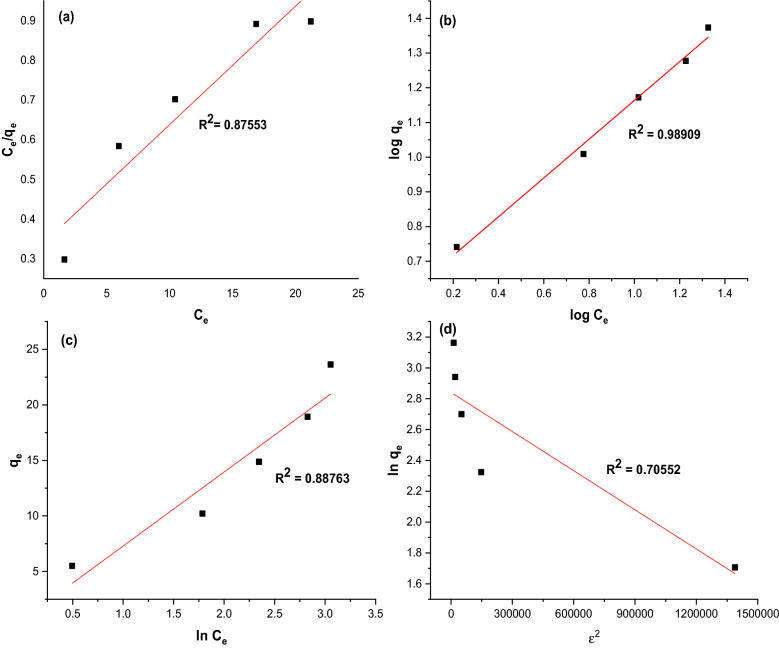
Plots of isotherm models for CR removal: (**a**) Langmuir, (**b**) Freundlich, (**c**) Temkin, and (**d**) Dubinin-Radushkevich.

The experimental plots and data predicted by four isotherm models (Langmuir, Freundlich, Temkin, and Dubinin-Radushkevich) in the form of the linearized equation are shown in Fig. 13 and Table 2, respectively. Generally, the correlation coefficient (R^2^) was used to indicate the best-fitting model. Based on the results, it can be deduced that the adsorption of CR was well-fitted with the Freundlich isotherm model since the R^2^ value was found to be 0.989, which is greater than the other isotherm models. This can be explained by the fact that GSC_WCO_ has a heterogeneous surface that can adsorb a multilayer of CR molecules. The heterogeneous adsorbent surface was known to have different types of adsorption sites for adsorbate to attach, with each site having different adsorption energy. According to the theory of Freundlich, the favorability of the adsorption process can be examined by indicating the adsorption intensity, $n$. The value of *n,* which lies between 1 and 10, was said to have a higher intensity of adsorption^70^. As depicted in Table 2, the value of $n$ for CR adsorption was 1.790, suggesting that the adsorption of CR onto the adsorbent was feasible and efficient. The surface of the adsorbent becomes more heterogenous when the slope value (1/*n*) is closer to zero^71^.

**Table 2:** Isotherm models parameter of CR adsorption onto GSC_WCO_.

Isotherm models	Parameters	Value
Langmuir	${Q}_{O}$ (mg/g)	33.50
	$b$ (L/mg)	0.088
	${R}_{L}$	0.363
	${R}^{2}$	0.876
Freundlich	${K}_{f}$ (mg/g)	4.025
	n	1.790
	${R}^{2}$	0.989
Temkin	${b}_{T}$ (J/mol)	6.664
	${A}_{T}$ (L/mg)	1.098
	${R}^{2}$	0.888
Dubinin-Redushkevich	${q}_{D}$	17.129
	${R}^{2}$	0.706

### Kinetic studies

**Figure 14:**
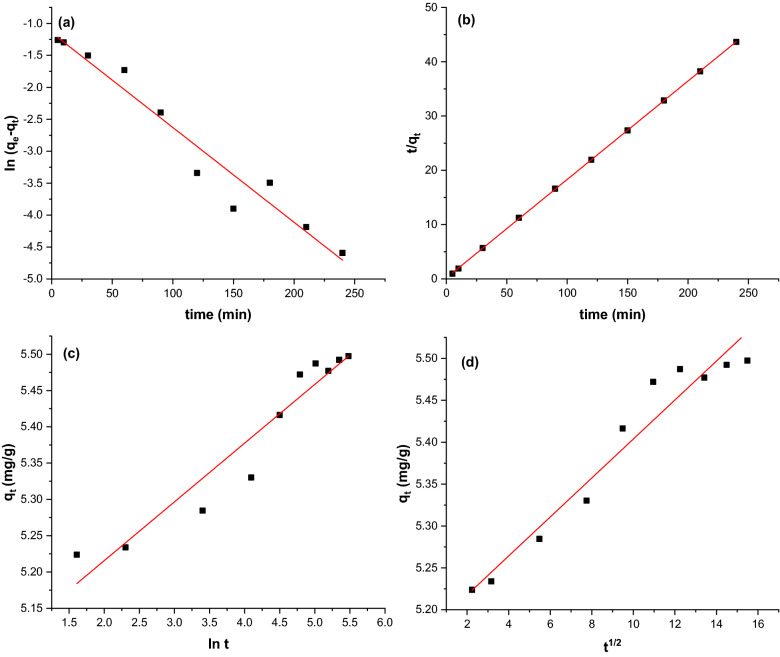
Plots of kinetic models for CR removal: (**a**) pseudo-first-order, (**b**) pseudo-second-order, (**c**) Elovich, and (**d**) intraparticle diffusion for CR adsorption onto GSC_WCO_.

Four kinetic models, namely pseudo-first-order, pseudo-second-order, Elovich, and intraparticle diffusion kinetic models, were fitted to the experimental data to understand the adsorption process mechanism better. Figure 14a–d show the plot of kinetic curve fittings of each kinetic model used in this study. The values of the parameters are summarized in Table 3. From the data obtained, the linearity of the plot suggests that the adsorption mechanism followed the pseudo-second-order kinetic model. As shown in Table 3, the R^2^ value of pseudo-second-order (R^2^ > 0.99) was closer to unity, indicating the adsorption of CR was operated through chemisorption. Chemisorption occurs when a chemical bonding is formed between the CR ions and GSC_WCO_. Besides, the calculated value of equilibrium adsorption capacity (q_e_) was closer to the experimental value (q_e,exp_) than the pseudo-first-order model. A similar result has been reported by Teow et al.^27^ using GSC from oil palm frond juice for the adsorptive removal of methylene blue.

**Table 3:** Kinetic parameters of CR adsorption onto GSC_WCO_.

Models	Parameters	Value
Pseudo-first-order	q_e_,_exp_ (mg/g)	5.51
	q_e_ (mg/g)	3.141
	k_1_ (min^−1^)	0.018
	R^2^	0.948
Pseudo-second-order	q_e_ (mg/g)	5.52
	${k}_{2}$ (g mg^−1^ min^−1^)	0.172
	R^2^	0.999
Elovich	α (mg/g min)	0.082
	β (g/mg)	12.358
	R^2^	0.917
Intraparticle diffusion	${k}_{id}$ (mg/g min^1/2^)	0.023
	C	5.171
	R^2^	0.941

### Thermodynamic study

The values of thermodynamic parameters, namely Gibb's free energy ($\Delta G^\circ )$, enthalpy ($\Delta H^\circ$), and entropy ($\Delta S^\circ$) changes of adsorption were obtained from the slope and y-intercept of linear plots of $ln {K}_{c}$ versus 1/T. As shown in Table 4, the negative value of $\Delta G^\circ$ obtained at various temperature ranges showed a highly feasible and spontaneous adsorption process without the involvement of an external energy source. The negative value of enthalpy change, $\Delta H^\circ$ indicated an exothermic nature of the CR dye adsorption process^72^. It was noted that the interaction behaviour between adsorbent and adsorbate solution could be well understood through $\Delta H^\circ$ magnitude. The negative value of $\Delta S^\circ$ (− 27.626) implies that the randomness was reduced at the adsorbent-adsorbate interface during the adsorption process^73^. The results clearly showed the trend of $\Delta G^\circ$ value increasing as the temperature increased from 298 to 313 K. This indicates the adsorption are highly favourable and occurred spontaneously. Hence, this process was more favoured at low than high temperatures.

**Table 4:** Thermodynamic parameters of CR adsorption onto GSC_WCO_.

Temperature (K)	$\Delta G^\circ$(KJ mol^−1^)	$\Delta H^\circ$(KJ mol^−1^)	$\Delta S^\circ$(KJ mol^−1^)
298	− 2.012	− 10.211	− 27.626
303	− 1.631		
313	− 1.464		

### Regeneration of GSC_WCO_

**Figure 15:**
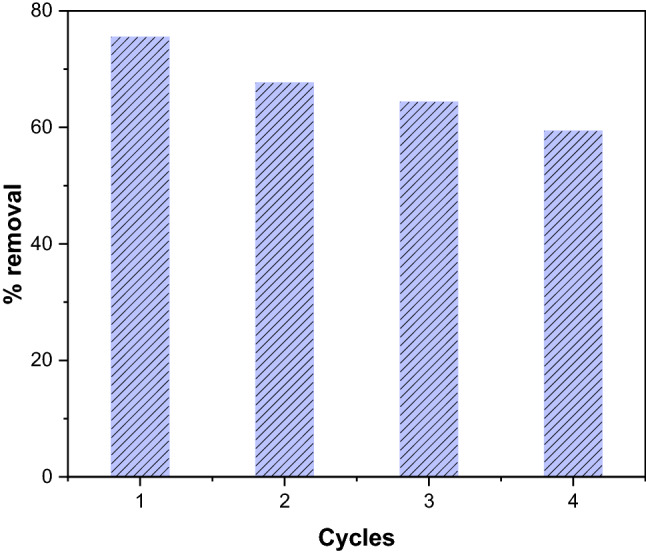
Regeneration study of GSC_WCO_ after four cycles.

Regarding environmental and economic perspectives, the regeneration of adsorbents is a crucial aspect that needs to be considered. The regeneration process would be beneficial in reducing the cost and the need for a new adsorbent. Moreover, the reusability of adsorbents can avoid the issue related to the disposal problem of used adsorbents. In this work, adsorption–desorption studies were performed for four consecutive cycles, and the removal efficiency after each cycle is shown in Fig. 15. The results showed that the removal efficiency for the 1st to 4th cycles decreased from 75.5 to 59.4%. Since chemisorption interaction occurred between CR molecules and adsorbent, the regeneration could not provide sufficient energy to break the bond between CR and GSC_WCO_, reducing efficiency after the 4th cycle^74^. Furthermore, the influence of desorbing agents also affected the desorption efficiency. It can be suggested that the desorption of GSC_WCO_ can be tested with other eluents such as HCl, acetone, or HNO_3_ to study its regeneration ability further. Given this, the regeneration capability of GSC_WCO_ can be considered acceptable since it can be reused up to several cycles.

### Performance of prepared GSC_WCO_

Table 5 compares this work's CR adsorption performance with other adsorbents from previously reported studies. The difference in CR capacities of the reported adsorbents was mainly attributed to the differences in adsorbent properties, including their porosity, surface area, functional groups, and experimental conditions. By comparison, the value of the adsorption capacity of the as-synthesized GSC was observed to be in good agreement with other adsorbents, indicating that CR could be easily adsorbed onto GSC_WCO_. This could be explained by the existence of graphene nanosheets on the sand surfaces, which provides several active adsorption sites on the composite adsorbent. Considering the cost production criteria, the utilization of waste cooking oil for developing GSC seems more economically beneficial than material that requires high resources.

**Table 5:** Comparison of adsorption capacities of CR by various adsorbents.

Adsorbents	Maximum adsorption capacity, mg/g	References
PVA/SA/ZSM‑5 zeolite membrane	5.33	^54^
Banana peel dust	1.73	^75^
Activated carbon-jujube seed	9.81	^76^
Activated carbon-coconut coir pith	6.72	^77^
Chitosan-coated quartz sand	3.56	^78^
Cashew nutshell	5.18	^79^
Montmorillonite	12.70	^80^
GSC_WCO_	5.52	This study

### Adsorption mechanism

**Figure 16:**
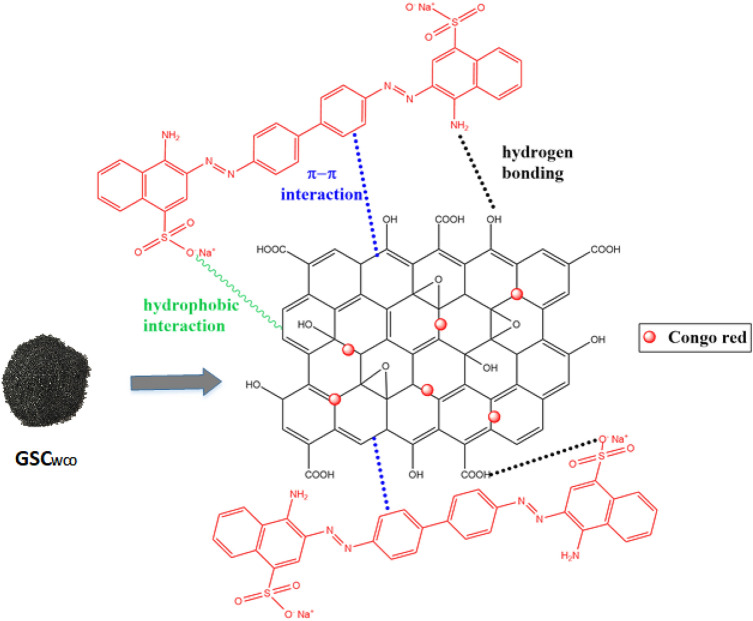
A proposed mechanism for the adsorption of CR on GSC_WCO_.

It is crucial to understand the adsorption mechanism of organic dye on the novel adsorbent of GSC_WCO._ Generally, sand possesses hydrophilic surfaces due to the presence of –OH groups on the surface of the particle^50^. The process of embedding graphene sheets on sand somehow provides physical support and enhances the accessibility of the binding sites. Due to its inherent hydrophobic nature, the sand surface properties were changed from hydrophilic to hydrophobic features and possessed a highly delocalized π-electron conjugated structure of graphene^26^. Figure 16 shows a plausible interaction factor that could be held responsible for CR dye adsorption. GSC_WCO_ adsorb dye molecules due to several contaminant-GSC_WCO_ interactions, including π–π interactions, hydrogen bonding, and hydrophobic interaction. The π–π interaction between GSC_WCO_ and the adsorbate plays a dominant role, contributing to the more significant adsorption of anionic dyes. The sp^2^-hybridized single-atom layer structure of graphene was reasonably responsible for this interaction and thus able to form π–π bonds with C=C double bonds or benzene ring of organic dye molecules^81^. Apart from that, hydrogen bonding between dye molecules and GSC_WCO_ also favoured overall adsorption. The GSC_WCO_ composite surface mainly consists of hydroxyl and carboxyl groups^35^. The strong hydrogen bonding connection was initially formed when the CR dye having amino and oxygen groups (electron acceptor) interacted with various oxygen functionalities on GSC_WCO_ (electron donor). At the same time, a hydrophobic effect exists between the GSC_WCO_ surface's tails and CR's hydrophobic components at basic pH.

**Figure 17:**
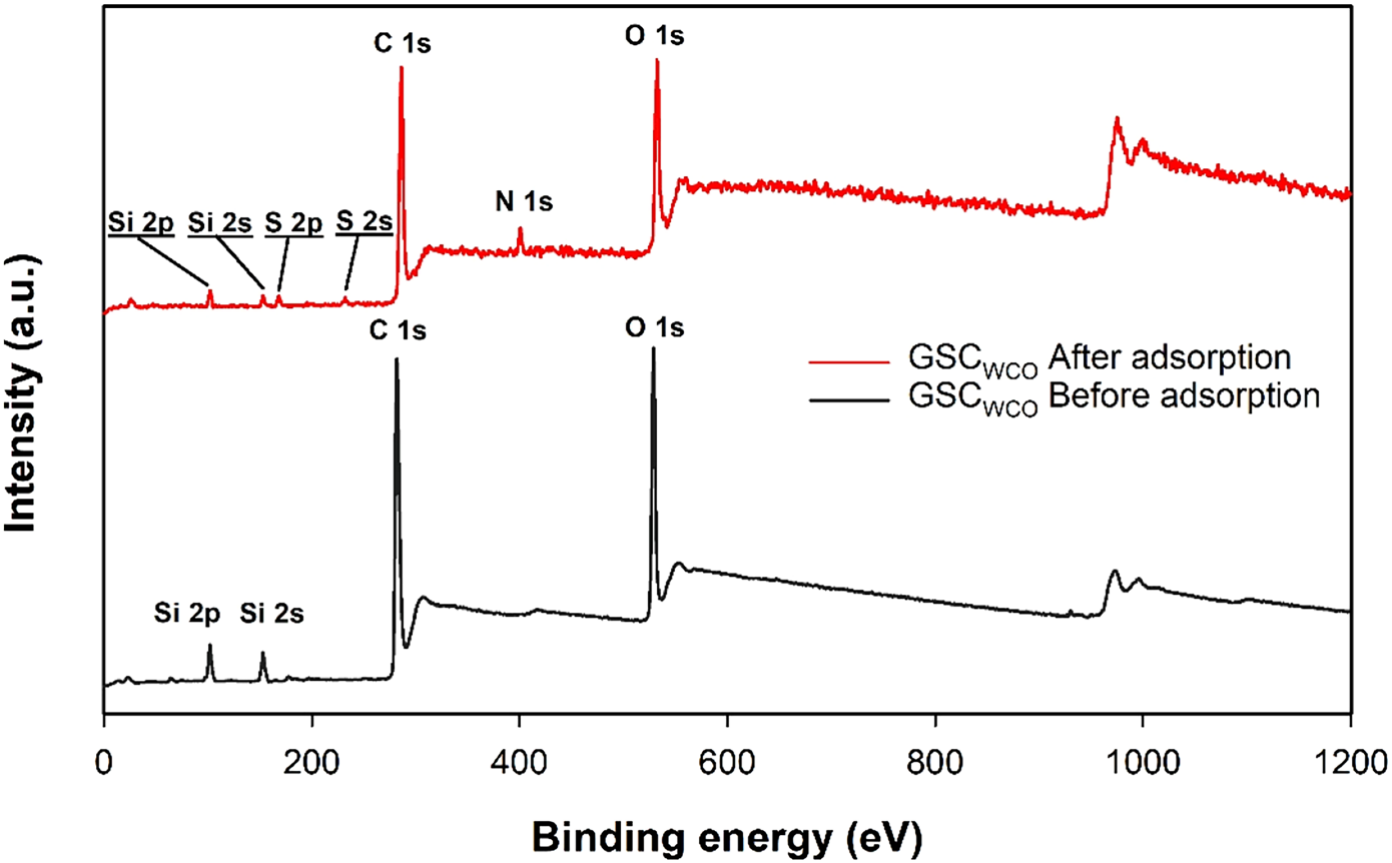
XPS survey spectra of GSC_WCO_ before and after adsorption.

Morphological features of GSC_WCO_ also had an essential role in the adsorption process. In “Characterization of GSC_WCO_” section, a series of characterizations (FTIR, XPS, Raman spectrum and SEM with EDX) were sufficient to confirm the multiple oxygen-containing functional groups on the surface of GSC_WCO_, which were responsible for adsorption. Moreover, an aggregated morphology and disappearance of pores were noticed from the post-adsorption characterization of GSC_WCO_ using SEM, indicating the interaction of adsorbent with CR. In addition, the full-scale XPS spectrum (Fig. 17) was utilized to inspect the bonding change on the surface of GSC_WCO_ before and after adsorption. As shown, the after-adsorption spectrum has some new peaks to differentiate it from the original GSC_WCO_. After adsorption, the peaks observed at 164 and 229 eV are related to S 2p and S 2s, respectively, attributed to the SO_3_^−^ group of CR. Further, 399 eV peak of N 1s was assigned to the N-containing functional groups of CR.

One common problem graphene faces is its tendency to aggregate^82^. However, the aggregation of graphene nanosheets can be prevented by attaching graphene to sand particles and further increasing its applicability to remove pollutants. Hence, graphene-coated sand prepared from WCO was potentially suitable for removing CR in an aqueous solution.

## Conclusion

A novel approach of synthesized graphene sand composite from waste cooking oil (GSC_WCO_) via a simple chemical approach was successfully reported in this study. WCO, as abundant and low-cost carbon precursors, was successfully converted to graphene sheets and coated on the sand surface. Raman and XRD analysis confirmed the formation of graphene-like structure materials. The SEM and EDX mapping showed that GSC_WCO_ had a rough and porous surface that can contribute to the greater efficient removal of pollutants. The applicability of GSC_WCO_ was tested for adsorptive removal of CR under optimal conditions (initial concentration of 20 mg/L, adsorbent amount of 1 g, contact time of 150 min). The sorption mechanism and rate of adsorption of GSC_WCO_ were better to fit with Freundlich and pseudo-second-order model, respectively, giving a maximum adsorption capacity of 5.53 mg/g at the temperature of 25 °C. The negative value of Gibb's free energy, enthalpy, and entropy changes confirmed sorption's feasibility, spontaneity, and exothermic nature. A reusability study revealed that GSC_WCO_ adsorbs CR up to four cycles with removal efficiency ranging from 75.5 to 59.4%. The π–π and hydrogen bonding interactions were postulated as the primary mechanism responsible for the adsorbent performance. Therefore, further development of graphene composite derived from carbon waste (WCO) would be an excellent potential for water pollution treatment and simultaneously help reduce the improper discharge of WCO.

## Supplementary Information

Supplementary Information.

## Data Availability

The authors confirm that the data used to support the findings are accessible within the article.
