# Supplementary material for: Sustainable synthesis of graphene sand composite from waste cooking oil for dye removal
Source: Sci Rep. 2023 Feb 2;13:1931. doi: 10.1038/s41598-023-27477-8 (PMC9894951; doi:10.1038/s41598-023-27477-8)
Supplement: Supplementary file 1 — Supplementary Information. [file 41598_2023_27477_MOESM1_ESM.docx]

**Supplementary information**

**Sustainable synthesis of graphene sand composite from waste cooking oil for dye removal**

Nor Syazwani Abdullah Sani,^1^ Wei Lun Ang,^1,2^ Abdul Wahab Mohammad,^3,1^ Alireza Nouri^1^ and Ebrahim Mahmoudi,^1,2*^

^1^Department of Chemical and Process Engineering, Faculty of Engineering and Built Environment, Universiti Kebangsaan Malaysia, 43600 Bangi, Selangor, Malaysia.

^2^Centre for Sustainable Process Technology (CESPRO), Faculty of Engineering and Built Environment, Universiti Kebangsaan Malaysia, 43600 Bangi, Selangor, Malaysia.

^3^Chemical and Water Desalination Engineering Program, College of Engineering, University of Sharjah, Sharjah, 27272, United Arab Emirates

*Corresponding author: E. Mahmoudi email: [mahmoudi.ebi@ukm.edu.my](mailto:mahmoudi.ebi@ukm.edu.my)

***S-1. Preparation of synthetic CR dye solution***

A 1000 mg L^-1^ stock of synthetic dye solution was made by weighing 1 g of dye powder and dissolving in one liter of distilled water. The solutions of desired concentrations were prepared by dilution from the stock solution for further experiments.

***S-2. Characterization of GSC_WCO_***

The morphological structures and elemental composition of river sand and GSC_WCO_ were analyzed by Field Emission Scanning Electron Microscope (FESEM, Supra 55VP, Zeiss, Germany) equipped with Energy Dispersive X-Ray (EDX, Oxford INCA, UK) and Transmission Electron Microscopy (TEM, Thermo Fisher, US). The crystallinity of the samples was determined with X-Ray Diffraction (XRD, Bruker D8 Advance, Germany) equipped with CuKα radiation and performed in the range 2*θ* of 5–80°. Raman spectra were carried out in the range of 500-3400 cm^-1^ by using confocal Micro-Raman Imaging Spectroscopy (DXR2xi, Thermo Scientific, US) equipped with a 532 nm laser as the light source, whereas Fourier Transforms Infrared Spectroscopy (FTIR, Nicolet 6700, Thermo Scientific, US) was used to determine the functional groups on the GSC_WCO_ surface. The spectrum was acquired in the range of 500-4000 cm^-1^ at a resolution of 4 cm^-1^ by using the KBr method in 1:10 proportion. Fresh cooking oil and WCO were also analyzed in the same range by using FTIR-ATR. The surface area of GSC_WCO_ was measured using N_2_ adsorption-desorption isotherm analysis (Micromeritics ASAP 2010, UK) at 77 K. The sample was degassed at 110℃ before being analyzed, and the surface area can be calculated using the BET equation. The surface charge of GSC_WCO_ composite was determined using the Zeta Sizer (Nano-ZS, Malvern Instrument Ltd, UK). The surface chemistry of GSC_WCO_ before and after adsorption were measured by X-ray photoelectron spectroscopy (XPS, Kratos Analytical Ltd, UK).

***S-3. Isotherm model studies***

The adsorption isotherm refers to the relationship between the number of molecules adsorbed at equilibrium and the adsorbate concentration at a constant temperature. In this study, several isotherm models such as Langmuir ^1^, Freundlich ^2^, Temkin, and Dubinin-Radushkevich ^3^ were utilized to fit the experimental data. The mathematical description (linearized form) of each isotherm model was summarized in Table 1.

**Table S1**: Isotherm models equation.

| Isotherms | Linear equation | Plot |
| --- | --- | --- |
| Langmuir | $\frac{C_{e}}{q_{e}}= \frac{1}{bQ_{O}}+ \frac{C_{e}}{Q_{o}}$ | $\frac{C_{e}}{q_{e}} vs C_{e}$ |
| Freundlich | $\log q_{e}=\log K_{f}+\frac{1}{n}\log C_{e}$ | $\log q_{e} vs \log C_{e}$ |
| Temkin | $q_{e}=\frac{RT}{b_{T}}\ln A_{T}+ \left( \frac{RT}{b_{T}} \right)\ln C_{e}$ | $q_{e} vs ln C_{e}$ |
| Dubinin-Radushkevich | $\ln\left( q_{e} \right)=\ln q_{D}- \beta\varepsilon^{2}$  $\varepsilon=RT ln(1+ \frac{1}{C_{e}}$)  $E=\frac{1}{\surd2\beta}$ | $\ln\left( q_{e} \right) vs \varepsilon^{2}$ |

where $q_{e}$ (mg/g) is the amount of CR adsorbed at equilibrium, $C_{e}$ (mg/L) is the equilibrium adsorbate concentration in solution, while $Q_{O}$ (mg/g) is the maximum adsorption capacity, $b$ (L/mg) is Langmuir adsorption constant, $K_{f}$ (mg/g) is known as Freundlich constant, which corresponds to adsorption capacity, $n$ is an adsorption intensity, $A_{T}$ is termed as Temkin isotherm equilibrium binding constant (L/mg), $b_{T}$ is the Temkin constant (J/mol), $R$ is a universal gas constant (8.314 J mol^-1^K^-1^), $q_{D}$ Indicates the theoretical isotherm saturation capacity (mg/g), $\beta$ denotes the isotherm constant of sorption energy (mol/K^2^J^2^), and $\varepsilon$ is Polanyi potential.

***S-4. Kinetic model studies***

Studies on the kinetic model were useful in explaining the controlling mechanism of adsorption and the potential of the rate-controlling step ^4^. To analyze the CR adsorption onto GSC_WCO,_ kinetic models of pseudo-first-order, pseudo-second-order, Elovich, and intraparticle diffusion were evaluated based on the linearized equation represented in Table 2.

**Table S2:** Kinetic models equation.

| Kinetic models | Linear equation | Plot |
| --- | --- | --- |
| Pseudo-first order (Lagergreen) | $\ln(q_{e}-q_{t})=\ln q_{e} -k_{1}t$ | $\ln(q_{e}-q_{t})vs t$ |
| Pseudo-second order | $\frac{t}{q_{t}}=\frac{1}{k_{2}q_{e}^{2}}+\frac{t}{q_{e}}$ | $\frac{t}{q_{t}}vs t$ |
| Elovich | $q_{t}=\frac{1}{\beta}\ln(\alpha\beta)+ \left( \frac{1}{\beta} \right)\ln t$ | $q_{t} vs ln t$ |
| Intraparticle diffusion | $q_{t}=k_{id} t^{0.5}+ C$ | $q_{t} vs t^{0.5}$ |

where $q_{e}$ and $q_{t}$ are defined as the amount of CR adsorbed at equilibrium (mg/g) and at time t (min), respectively, $k_{1}$(hr^-1^), $k_{2}$(g mg^-1^ h^-1^) and $k_{id}$ (mg/g min^1/2^) represents the rate constant of pseudo-first-order, pseudo-second-order, and intraparticle diffusion models, respectively, $\alpha$ (mg/(g min) is the initial adsorption rate, $\beta$ (g/mg) is the desorption constant, and C is the intercept.

***S-5. Thermodynamic studies***

The thermodynamic of the adsorption process was investigated based on the estimation of thermodynamic parameters such as standard Gibbs free energy change, ΔG˚ (kJ/mol), change in enthalpy ΔH˚ (kJ/mol), and entropy change ΔS˚ (J mol^-1^ K^-1^). These parameters helped in determining whether the adsorption process was exothermic, endothermic, spontaneous, random or the temperature of the process was favorable or not. All the parameters were calculated at different temperatures according to Equation (1), Equation (2), and Equation (3), respectively.

$\Delta G=-RT\ln K_{c}$……………………………………(1)

$\ln K_{c}=\frac{\Delta S^{\circ}}{R}-\frac{\Delta H^{\circ}}{RT}$……………………………………..(2)

$K_{c}=\frac{q_{e}}{C_{e}}$……………………………….......................(3)

where $R$ is the universal gas constant (8.314 J mol^-1^ K^-1^), $T$ is the temperature in Kelvin, $K_{c}$ is the equilibrium constant.

***S-6. Regeneration and reusability***

The regeneration study for CR dyes was conducted by immersing 1 g of CR-loaded GSC_WCO_ in desorbing agent NaOH (0.1 M) and continuously stirred for 24 hr at room temperature. The desorbed equilibrium concentration (C_D_) was recorded. The adsorbent was then rinsed with ultra-pure water, dried, and reused for subsequent cycle ^5^.

***S-7. Calculation of number of graphene layer (n)***

The interlayer spacing can be calculated by using Bragg's law equation:

$d=n\lambda/(2\sin\theta)$ (4)

where, d is an interplaner spacing or d-planar (in Å), λ is the wavelength of incident X-ray (1.5406 Å), θ is the peak position (in radian), and n is an order of diffraction (n=1).

XRD diffraction can be used to determine the crystallize size by using Scherrer's method formulated in Equation (2) ^6^.

$D=K\lambda/\beta\cos\theta$ (5)

where, K is a constant shape crystallite factor (0.9), λ is the wavelength of the X-ray, $\beta$ is half width of the diffraction band (FWHM in radian), and θ is the diffraction angle (peak position in radian).

The average number of graphene layer (n) can be calculated based on the combination equation of Debye-Scherrer and Bragg's law and can be expressed as:

$n=D/d+1$ (6)

**References**

1. Irving Langmuir. The adsorption of gases on plane surfaces of glass, mica and platinum. *J. Am. Chem. Soc.* 1361–1403 (1918). doi:10.1021/ja01269a066

2. Freundlich, H. Über die Adsorption in Lösungen. *Zeitschrift für Phys. Chemie* **57U**, 385–470 (1907).

3. Iftekhar, S., Ramasamy, D. L., Srivastava, V., Asif, M. B. & Sillanpää, M. Understanding the factors affecting the adsorption of Lanthanum using different adsorbents: A critical review. *Chemosphere* **204**, 413–430 (2018).

4. Abbas, M. Experimental investigation of titanium dioxide as an adsorbent for removal of Congo red from aqueous solution, equilibrium and kinetics modeling. *J. Water Reuse Desalin.* **10**, 251–266 (2020).

5. Ahmad, R. & Ansari, K. Comparative study for adsorption of congo red and methylene blue dye on chitosan modified hybrid nanocomposite. *Process Biochem.* **108**, 90–102 (2021).

6. Fatimah, S. *et al.* ASEAN Journal of Science and Engineering How to Calculate Crystallite Size from X-Ray Diffraction ( XRD ) using Scherrer Method. **2**, 65–76 (2022).
